# Obesity-Related Communication in Digital Chinese News From Mainland China, Hong Kong, and Taiwan: Automated Content Analysis

**DOI:** 10.2196/26660

**Published:** 2021-11-23

**Authors:** Angela Chang, Peter Johannes Schulz, Wen Jiao, Matthew Tingchi Liu

**Affiliations:** 1 Faculty of Social Sciences University of Macau Taipa Macao; 2 Institute of Communication and Health University of Lugano Lugano Switzerland; 3 Faculty of Business Administration University of Macau Taipa Macao

**Keywords:** public health, computational content, digital research methods, obesity discourse, gene disorders, noncommunicable disease

## Abstract

**Background:**

The fact that the number of individuals with obesity has increased worldwide calls into question media efforts for informing the public. This study attempts to determine the ways in which the mainstream digital news covers the etiology of obesity and diseases associated with the burden of obesity.

**Objective:**

The dual objectives of this study are to obtain an understanding of what the news reports on obesity and to explore meaning in data by extending the preconceived grounded theory.

**Methods:**

The 10 years of news text from 2010 to 2019 compared the development of obesity-related coverage and its potential impact on its perception in Mainland China, Hong Kong, and Taiwan. Digital news stories on obesity along with affliction and inferences in 9 Chinese mainstream newspapers were sampled. An automatic content analysis tool, DiVoMiner was proposed. This computer-aided platform is designed to organize and filter large sets of data on the basis of the patterns of word occurrence and term discovery. Another programming language, Python 3, was used to explore connections and patterns created by the aggregated interactions.

**Results:**

A total of 30,968 news stories were identified with increasing attention since 2016. The highest intensity of newspaper coverage of obesity communication was observed in Taiwan. Overall, a stronger focus on 2 shared causative attributes of obesity is on stress (n=4483, 33.0%) and tobacco use (n=3148, 23.2%). The burdens of obesity and cardiovascular diseases are implied to be the most, despite the aggregated interaction of edge centrality showing the highest link between the “cancer” and obesity. This study goes beyond traditional journalism studies by extending the framework of computational and customizable web-based text analysis. This could set a norm for researchers and practitioners who work on data projects largely for an innovative attempt.

**Conclusions:**

Similar to previous studies, the discourse between the obesity epidemic and personal afflictions is the most emphasized approach. Our study also indicates that the inclination of blaming personal attributes for health afflictions potentially limits social and governmental responsibility for addressing this issue.

## Introduction

### Background

The prevalence of obesity has increased worldwide, including China, the world’s most populous country. A recent report on China indicated that 46.4 million women (14.9%) and 43.2 million men (10.8%) are obese, which reflects a major health challenge [1]. Obesity not only represents a cosmetic concern but also is a medical problem that increases the risk of other diseases and health problems [2]. Obesity itself is a disease that can cause premature disability and death by increasing the risk of cardiovascular diseases, metabolic disease, musculoskeletal disease, osteoarthritis, dementia, depression, and some types of cancers [2,3]. Being obese can increase the risk of many potentially serious health conditions. Because of the complexity of diseases associated with obesity, it is one of the most difficult public health issues our society should deal with.

News plays an important role in distributing reliable information for general readers or subscribed peer-publics. The current body of literature has adopted the framing theory for reporting news content, which highlights some aspects of themes in news stories to help define ill health problems [4-6]. The most applied thematic coverage is associated with increased societal attributions, while episodic coverage is related to increased individualistic responsibility and punitive treatment [5,7,8]. Considering obesity a key public health priority, news stories that report on obesity may impact and induce shifts in readers’ perceptions of the obesity problem [4,5].

In this study, the obesity issue is addressed from two approaches: first, we face new concentrations of media power, leading to new inequalities and insecurities with respect to data geographies and different data-related practices [9]. Specifically, there are many nefarious motivations underlying the creation of disinformation by giving one major reason why a person or group might want to spread wrong information [6-8]. Previous nascent studies on media content have shown that computational methods facilitate digital journalistic data collection and metadata analysis [10-12]. Studies have shown the importance of machine computational algorithms in facilitating big data storage and analysis to empower beneficial information for extending the preconceived grounded theory [10,13].

Second, comparatively fewer studies on journalistic content analysis on health issues are in languages other than English [6]. Thus, 9 mainstream and digital journalistic content in Chinese with high browsing and circulation were examined. Our results may broaden the scope for learning public health information from news media in terms of languages and geographical comparison considerations.

### Goals of This Study

This study examined the causes and strength of inferences associated with obesity by parsing meaning from unstructured data to guide decisions. The dual objectives of this study are to understand how obesity is covered in news along with the timeline of obesity communication and what journalistic discourse says about etiology, afflictions issues, and diseases associated with the burden of obesity. We draw upon recent advances in computational text analysis to develop a hybrid approach to the deductive analysis of large scale of digital course [14].

The analysis consists of comparisons between the digital journalistic work in Mainland China and its 2 neighboring territories with the condensed Chinese population, Taiwan, and Hong Kong. The comparison is meaningful by considering different governmental paths and socioeconomic developments in the Chinese civilization. The news content also highlights the media environment and public communication priority which potentially impact readers’ perceptions of health care management.

### Research Questions

The conceptual foundations of machine computation to text classification were used. A strategy for theory-assisted (driven and computer) classification of large text corpora with established categories was evaluated. Given the natural discourse on digital media, it is argued whether linguistic preprocessing can improve classification quality. Therefore, 6 research questions have been raised for studying the trend and patterns of obesity communication and mediated associations of obesity coverage for web-based readers:

1. What is the trend of coverage of the obesity epidemic and how has the pattern of digital journalistic stories developed over time?
2. What etiological factors are associated with obesity covered in the news?
3. What types of diseases linked with obesity are presented in the news coverage?
4. What type of etiology and disease linked with obesity have been ignored?
5. What is the strength of the correlation of observable variables (etiology and disease) linked with the burden of obesity by considering the total effect (a combination of direct and indirect variables)?
6. What type of message frames (thematic or episodic) does health-related news employ?

## Methods

### The Approach of Automated Content Analysis

The study protocol included defining text documents of news by retrieving web-based sources and testing the depth and scale of the data. Previous studies emphasized the importance of combining computational and manual techniques to preserve the strengths of traditional content analysis and the innovative large-scale capacity of big data analysis [6,15,16]. Traditional content analysis provides systematic rigor and contextual sensitivity while the computational analysis maximizes the algorithmic accuracy of computational methods to yield more favorable results [17]. Thus, an approach of combining computational and manual methods throughout the data analysis process was used.

Machine computational algorithms were used with a proposed platform, DiVoMiner. The tool of text processing is a modern technique for automated content analysis. It has been designed to organize and filter large archives of documents on the basis of patterns of word co-occurrence and term discovery [16,18]. Specifically, DiVoMiner is a computational platform that integrates functions of data cleaning, filtering, analysis, and visualization from the perspective of social sciences. It is an operating system that allows users to monitor the data information and provide content analysis in a timely manner.

Automated content analysis to identify prominent issues has become a commonly used methodology, particularly in communication sciences [19-23]. The computational procedure began with customized news data preparation, storage, preprocessing, screening, and cleaning. The process of data filtering is imperative and important for organizing unstructured data to be processed in a manner to be further analyzed with validity [9,11]. The use of a computational algorithm facilitates detecting terms and topic-modeling while focusing on theory development and application [24-27].

**Figure 1:**
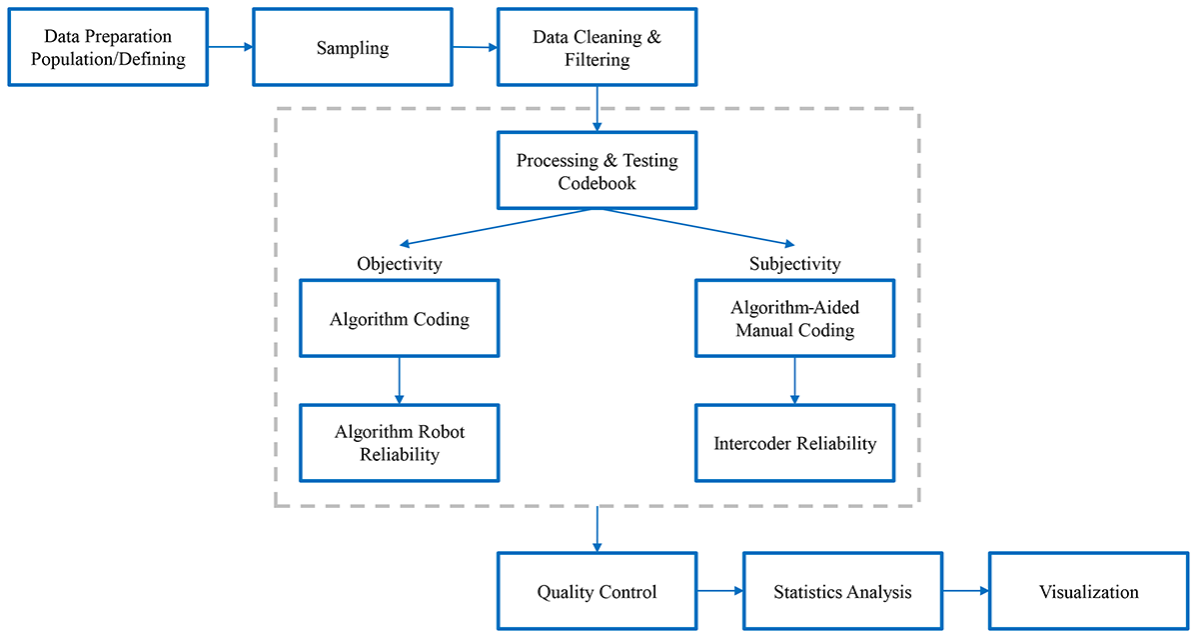
The workflow of hybrid and automatic content analysis for computer-assisted classification of DiVoMiner.

One of the tasks was to make news articles more discoverable. Therefore, each article that contained a keyword, a phrase in a sentence, or an assertion about obesity were sampled and screened. The process involved pilot coding, subsequent modification, and double coding, which could improve coding efficiency and data quality [27,28]. Figure 1 shows the workflow of hybrid and automatic content analysis for computer-assisted classification of DiVoMiner.

### Sampling News Media and Articles

The sampling criteria of digital journalistic work were in accordance with those of previous studies by considering the following three criteria: high circulation of news media, accessible with digital archives, and from the most populated cities with a severe obesity problem [6,7,16,28]. However, the newspapers were selected not only for a representation of what the mainstream newspapers offer its web-based readers but also for reasons in representing government-oriented (owned or business) news.

For example, 3 sampled newspapers in Mainland China focus on uncovering the potential impact of state influence on news production. It can be considered the mouthpiece of the government. Nevertheless, relative to state-owned and controlled media, business-oriented news agencies from Taiwan and Hong Kong are sampled to learn their editorial operation, which may fit into their desired investment profile [29,30]. The data span from January 1, 2010, to December 31, 2019, which would help understand the journalistic surveillance of obesity coverage for a decade.

Overall, 9 newspapers were selected: *Southern Metropolis Daily* (南方都市報), *Guangzhou Daily* (廣州日報), and *Beijing Evening News* (北京晚報) from Mainland China; *United Daily News* (聯合報), *Times Daily News* (中國時報), and *Liberty Times* (自由時報) from Taiwan; and *Apple’s Daily News* (蘋果日報), *Oriental Daily* (東方日報), and *MingPao* (明報) from Hong Kong.

### Keywords Developed for Automated Coding

The procedure of determining keywords included collecting and extracting words into the meaning group by the pkuseg—python. Thus, the word segmentation toolkit provides acceptable accuracy to meet the requirements of mediated associations in this study [16,28]. However, it is worth noting that Chinese words and words in the meaning group have a unique morphological system by selecting different settings on semiprefixes or semisuffixes [6,16]. For instance, the keywords related to an individual’s weight included “obese” (過肥) or “overweight” (過重); multiple terms describing obesity included “too big” (太胖) or “excessive weight” (體重超標); the semiprefix “too” (太) denotes the level of obesity for “too fat” (太肥) or “too heavy” (太重); the semisuffix “-zi” (子) is able to nominalize an adjective “fat” (胖) into “pang-zi” (a fat man) (胖子). Filters employed word segments such as obesity, obesity-related keywords, and alternative terms to create a logical phrase allowing search results to be more objective. The objectivity of journalistic data should not be viewed as an absolute, but rather as a performance that is grounded in practice.

Many Chinese lexicons for covering obesity and its causal link seem to be interchangeable in the codebook, despite the word “cause” being typically used to express how one factor assumes responsibility for its effects, while “lead to” is used to express the result of a certain action or event. Therefore, a tailored codebook containing keywords, phrases, causal link, etiological inference, and diseases potentially related to obesity has been developed for obesity-related communication from a journalistic perspective. The codebook was tested several times along with manual annotations in a fast, uncomplicated, but more efficient and satisfying way.

The drafted codebook consists of 13 target words, 13 etiologies, and 7 categories of noncommunicable diseases (NCDs) adapted from previous studies [2,3,6,28]. However, it was decided that any article that contained the keyword “obesity” and its related terms a minimum of 2 times was ideal to be considered valid samples. The threshold was decided in a data-driven manner because the existing supervised algorithms did not yield output variables satisfactorily. News articles that contained related terms at least 2 times can ensure valid terms. This also allowed researchers to uncover associations that could not have been found using analog research methods [30]. Additionally, objectivity is a core journalistic norm, which involves collecting and disseminating verifiable facts delivered in a detached manner [29,30]. Therefore, each article containing the keyword more than 1 time is viewed as evidence to lend weight to assertions and increase objectivity in news reports.

Our results include 11 target words, 11 etiologies, and 4 disease categories specifically related to obesity coverage. The revised codebook sustains the framing of obesity disorder as an NCD in the Chinese newspapers. A list of target words for obesity, causal link–related terms, and obesity with etiology and disease in Chinese with English translations is displayed in Multimedia Appendix 1.

### Data Preprocessing

To ensure the validity and reliability of machine analysis, a manual procedure was used to ensure a more reliable computational process for the generated text, including frequent terms, topics, and causal links [31]. Therefore, 5 Chinese graduate students majoring in communications and new media were trained for over 10 hours to act as independent coders. Each coder was required to code 500 sampled with 20% of overlapping news stories by providing dichotomous judgments and to indicate uncertainty. If a news article provided a verbal statement that suggested a problem associated with excess weight, it was coded for the presence of a news claim. Coders discussed all disagreements and uncertainties with the first author during the final stage of the analysis.

Previous communication studies have recommended 80% agreement between coders as an acceptable minimum [6,14,16]. The reliability for each variable in this study indicated substantial agreement across coders (Cohen κ= 0.81-1.00; *P*<.001; 95% CI 0.704-0.908), indicating high confidence in the reliability of the test data. Taken together, previous studies guided the conceptualization of mediated association to capture explicit assertions of casual links [11,26,32]. Analogical reasoning on linguistic regularities for developing a more comprehensive and reliable association is imperative.

### Etiology of Causality Assessment

Causality is one of the discourse analyses performed by extracting semantic relationships between cause and consequence for learning and predicting the structure of a sentence [33]. Analyzing causality is a common practice for studying the data corpus of machine learning [6,26]. Considering the widespread use and the complex nature of the Chinese language, DiVoMiner helped implement the taxonomy to measure the concept of causality. Specifically, based on the internal rules of mining text [31,34,35], DiVoMiner guided the machine to automatically label obesity and its related keywords. The classification results of algorithm coding generated by DiVoMiner were obtained simultaneously.

For correlation and total effect analysis, Python3 NetworkX was used to examine a network. The concept of obesity-related terms was considered as nodes while the article numbers were considered as weights. Betweenness centrality is the sum of the fraction by considering the shortest paths of all pairs in a network [36]. The greater edge centrality (EC) indicates that the terms are more connected. To explore the strength of the association between 2 variables, the width of an edge was calculated for each node. For instance, a node of “high calorie” shows high strength with the use of “heart attack.” Several examples of conceptual terms analyzed by the DiVoMiner are shown in Multimedia Appendix 2.

## Results

### Codebook and Data Trend

In total, 13 target words, 11 etiologies, and 4 categories of disease were closely related to obesity reports, which sustains the framing of obesity disorder as an NCD in the Chinese newspapers. Based on a contrast analysis of semantics developed, the episodic theme emphasized 6 variables, including an individual’s behavior related to stress, tobacco use, overuse of alcohol, decreased physical activity, improper diet, and drug use. In comparison, the thematic theme included 5 variables including genetic disorders, environmental pollution, family status, poor economy, as well as social and economic systems. Four main types of diseases such as cardiovascular, metabolic, cancer, and autoimmune were identified as major pathogenic links with obesity in the news.

**Figure 2:**
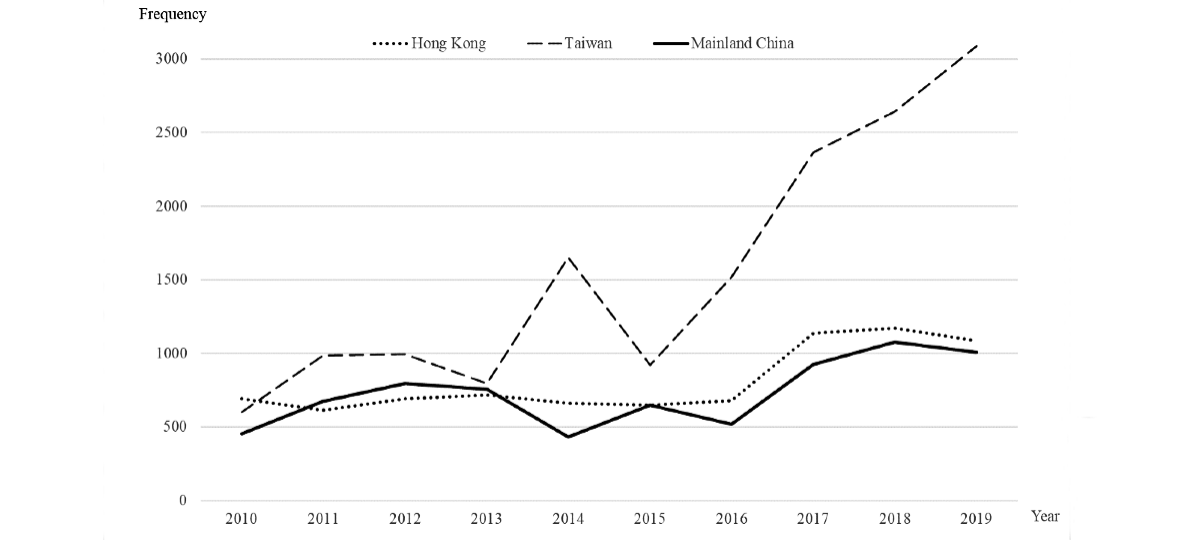
Trend and development of obesity with causal claims in news articles in Mainland China, Hong Kong, and Taiwan from 2010 to 2019.

In all, 30,968 articles covering obesity along with the etiology of causal inference were identified from 2010 to 2019, which show a clear trend of increasing attention over time. To facilitate comparisons across media development process and cultures, the numbers and ranks of obesity-related articles were significantly different between Mainland China and the neighboring areas in Hong Kong and Taiwan (*χ*^2^_30_=1098.88, *P*<.001). The highest surge in obesity-related coverage in Mainland China was observed in 2018 and 2019, but it showed a big decrease in coverage in 2014 and 2016. Figure 2 shows an overview of the trend and development of obesity with causal claims in the news articles in Mainland China, Hong Kong, and Taiwan from 2010 to 2019.

An overall analysis revealed that news articles related to the obesity epidemic peaked in 2019 (n=5183, 16.7%), followed by 2018 (n=4890, 15.8%) and 2017 (n=4432, 14.3%). The average coverage of obesity and its etiology and related diseases was 3097 articles per year. In comparison, above-average reports were observed in Taiwan’s journalistic discourse. A trend of below-average reporting was found from within the years between 2010 and 2017 (range from 1746 to 2744 articles). The intensity of journalistic coverage of the obesity epidemic peaked in Taiwan (n=15,571), followed by newspapers in Hong Kong (n=8103) and Mainland China (n=7294). Table 1 displays the distribution of obesity-related news articles in Chinese newspapers in Mainland China, Hong Kong, and Taiwan from 2010 to 2019.

**Table 1:** Distribution of obesity-related news articles in Chinese newspapers in Mainland China, Hong Kong, and Taiwan from 2010 to 2019.

Year	Obesity-related news articles, n (%)			
	Mainland China (n=7294)	Taiwan (n=15,571)	Hong Kong (n=8103)	All (n=30,968)
2010	453 (6.2)	602 (3.9)	691 (8.5)	1746 (5.6)
2011	673 (9.2)	985 (6.3)	616 (7.6)	2274 (7.3)
2012	798 (10.9)	994 (6.4)	693 (8.6)	2485 (8.0)
2013	759 (10.4)	797 (5.1)	716 (8.8)	2272 (7.3)
2014	431 (5.9)	1652 (10.6)	661 (8.2)	2744 (8.9)
2015	650 (8.9)	922 (5.9)	650 (8.0)	2222 (7.2)
2016	519 (7.1)	1520 (9.8)	681 (8.4)	2720 (8.8)
2017	927 (12.7)	2367 (15.2)	1138 (14.0)	4432 (14.3)
2018	1077 (14.8)	2643 (17.0)	1170 (14.4)	4890 (15.8)
2019	1007 (13.8)	3089 (19.8)	1087 (13.4)	5183 (16.7)

### Etiology of Obesity-Related Communication

The etiological attributes of obesity coverage were presented in 16,991 of 30,968 (54.9%) articles initially collected. The highest intensity of newspaper coverage on obesity-mediated causes was observed in Taiwan (n=8090, 47.6%), followed by Mainland China (n=5584, 32.9%) and Hong Kong (n=3317, 19.5%). Overall, there was a substantially stronger focus on 3 shared causative inferences of obesity such as stress (n=4483, 26.4%), tobacco use (n=3148, 18.5%), and genes (n=2418, 26.4%).

In comparison, one common causal attribute of obesity, social and economic system (n=7, 0.2%) received the least coverage in all newspapers. One etiology of causative conditioning, such as drug use, also received scarce coverage in Mainland China (n=47, 1.1%), while one causative agent of the poor social economy also received scarce coverage in Taiwan (n=24, 1.6%) and Hong Kong (n=12, 2.1%). In brief, the numbers and ranks of the shared causes of obesity between controlled news media and business-oriented news were significantly different (*χ*^2^_33_=348.48, *P*<.001).

The pattern of using episodic (personal) versus thematic (nonpersonal) agents in 3 areas is similar. Among a total of 16,991 articles, the episodic risk factor makes up the majority of causes of obesity, which were covered (n=13,587, 80.0%), compared to the influence of thematic conditions (n=3404, 20.0%). Specifically, the number of episodic themes associated with obesity were the highest in Taiwan (n=6563, 48.3%), followed by Mainland China (n=4283, 31.5%) and Hong Kong (n=2741, 20.2%). Etiological factors including stress, tobacco use, and alcohol overuse were strongly associated with obesity coverage in all newspapers.

On contrast analysis of semantic concepts, the number of thematic themes associated with obesity were also the highest in Taiwan (n=1527, 44.9%), followed by Mainland China (n=1301, 38.2%) and Hong Kong (n=576, 16.9%). Genetics contribute to 61.6%-80.7% of obesity with further news coverage. Table 2 shows a comparison of causal attributes of obesity in the news articles under 2 themes in Mainland China, Hong Kong, and Taiwan from 2010 to 2019.

**Table 2:** Comparison of causal attributes of obesity in the news articles under 2 themes in Mainland China, Hong Kong, and Taiwan from 2010 to 2019.

Causal attributes of obesity			News articles, n (%)						
			Mainland China		Taiwan		Hong Kong		All
**Episodic theme**			4283 (100)		6563 (100)		2741 (100)		13,587 (100)
	Stress	1523 (35.6)		2046 (31.2)		914 (33.3)		4483 (33.0)	
	Tobacco use	944 (22.0)		1635 (24.9)		569 (20.8)		3148 (23.2)	
	Alcohol overuse	863 (20.1)		1121 (17.1)		430 (15.7)		2414 (17.8)	
	Lack of physical activity	500 (11.7)		1102 (16.8)		552 (20.1)		2154 (15.9)	
	Improper diet	406 (9.5)		590 (9.0)		262 (9.6)		1258 (9.3)	
	Drug use	47 (1.1)		69 (1.1)		14 (0.5)		130 (1.0)	
**Thematic theme**			1301 (100)		1527 (100)		576 (100)		3404 (100)
	Genetic disorders	801 (61.6)		1152 (75.4)		465 (80.7)		2418 (71.0)	
	Environmental pollution	348 (26.7)		278 (18.2)		76 (13.2)		702 (20.6)	
	Family status	63 (4.8)		71 (4.6)		19 (3.3)		153 (4.5)	
	Poor societal economy	88 (6.8)		24 (1.6)		12 (2.1)		124 (3.6)	
	Social and economic system	1 (0.1)		2 (0.1)		4 (0.7)		7 (0.2)	

There were 4 main types of diseases including cardiovascular, metabolic, cancer, and autoimmune diseases associated with obesity communication in the Chinese newspapers. Among 30,968 articles initially collected, the news coverage of diseases linked with obesity were presented in a total of 20,111 articles (64.9%). The highest intensity of news coverage of the obesity mediated diseases was observed in Taiwan (n=10,622, 52.8%), followed by Mainland China (n=5432, 27.0%) and Hong Kong (n=4057, 20.2%).

Three disease categories were paid the most attention for coverage: cardiovascular diseases (n=8477, 42.2%), metabolic diseases (n=6869, 34.2%), and cancer (n=4762, 23.7%). Autoimmune diseases received relatively scarce reports associated with obesity (n=3, 0.0%), while the others were totally ignored (eg, musculoskeletal or neurological disorder). The diseases associated with obesity also show a significant difference among the 9 newspapers (*χ*^2^_36_=83.74, *P*<.001). Table 3 displays the disease types associated with obesity in news articles in Mainland China, Hong Kong, and Taiwan from 2010 to 2019.

To examine the strength of disease etiology associated with obesity-related communication, a network analysis displayed a connection created by the aggregated interaction. As a result of the betweenness EC measurement, the rank-correlation between the causal effects and betweenness centrality was 0.17. Specifically, the aggregated interaction between the 2 terms “obesity” and “cancer” was the highest (EC=0.133), followed by the mediated relation between “obesity” and “metabolic diseases” (EC=0.030), and “obesity” and “cardiovascular diseases” (EC=0.024).

**Table 3:** Disease types associated with obesity in news articles in Mainland China, Hong Kong, and Taiwan from 2010 to 2019.

Disease types	Obesity-related news articles, n (%)			
	Mainland China (n=5432)	Taiwan (n=10,622)	Hong Kong (n=4057)	All (n=20,111)
Cardiovascular diseases	2306 (42.5)	4349 (40.9)	1822 (44.9)	8477 (42.2)
Metabolic diseases	1951 (35.9)	3593 (33.8)	1325 (32.7)	6869 (34.2)
Cancer	1175 (21.6)	2678 (25.2)	909 (22.4)	4762 (23.7)
Autoimmune diseases	0 (0.0)	2 (0.0)	1 (0.0)	3 (0.0)

**Figure 3:**
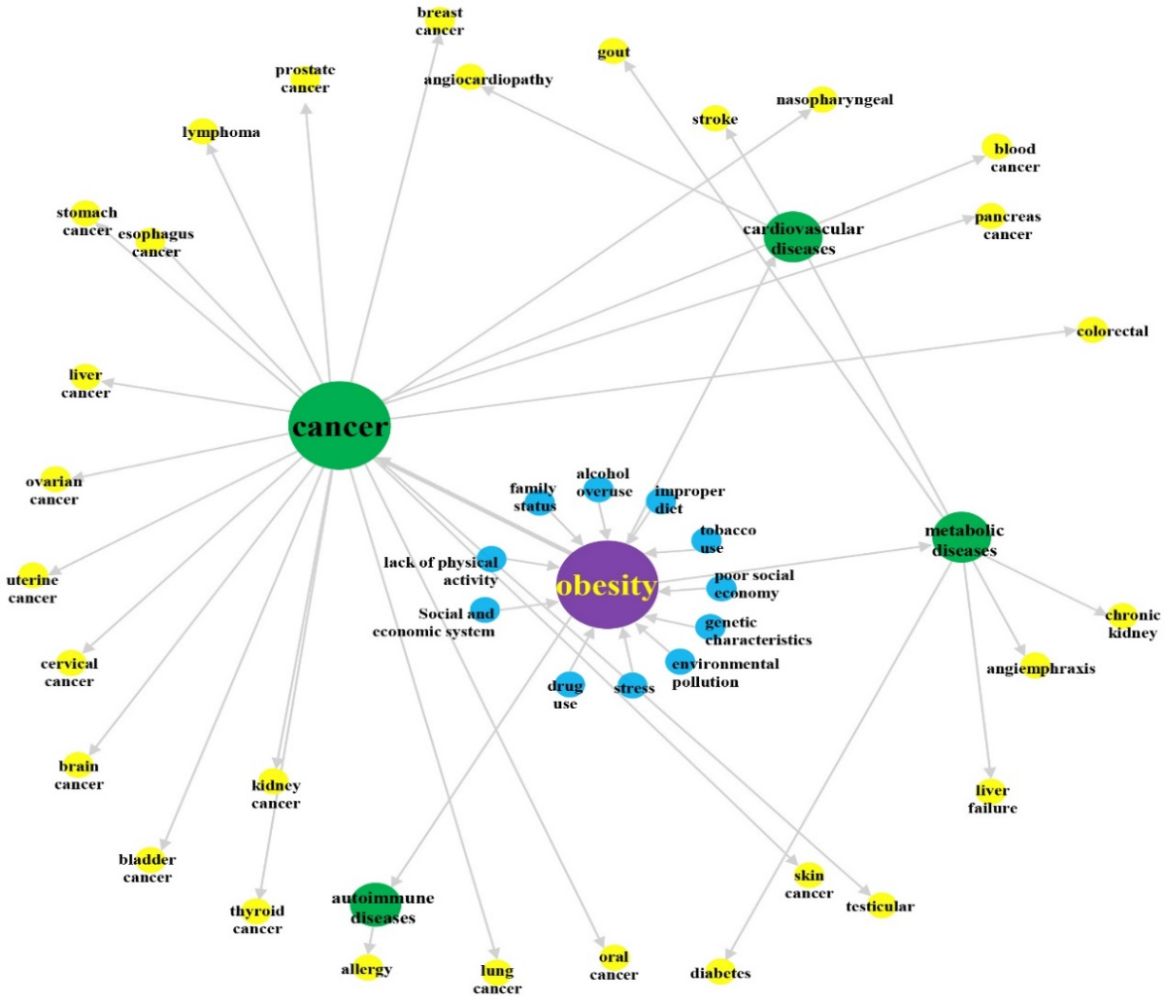
A direct graph color-coded on the basis of the betweenness centrality of each vertex from least (yellow) to greatest (blue) with regard to etiology and noncommunicable diseases associated with obesity in the news articles in Mainland China, Hong Kong, and Taiwan from 2010 to 2019.

The result shows that overweight and obesity are associated with an increased risk of 21 types of cancer and a total of 11 behavioral and environmental factors. An explanation for the different trends of the centrality measures as the network density increases is that renal cell cancer (RCC) is the major type of kidney cancer with increasing incidence; furthermore, obesity is one of the well-established risk factors for RCC, as covered in the news. In addition, several factors of an individual’s behavior such as poor diet and too little physical activity are highlighted, while factors of environmental variables including pollution and poor societal economy are observed. Figure 3 displays a direct graph color-coded on the basis of the betweenness centrality of each vertex from the least (yellow) to the greatest (blue) with regard to etiology and NCDs associated with obesity in the news articles in Mainland China, Hong Kong, and Taiwan from 2010 to 2019.

## Discussion

### Principal Findings

The high prevalence of obesity is associated with an enormous medical, social, and economic burden. Chinese news coverage on obesity along with etiology of causal inference shows a clear trend of increasing attention over time. This also suggests that chronic stress is strongly associated with obesity. The association between stress and obesity can lead to overeating of foods that are high in fat, sugar, and calories, which, in turn, can lead to weight gain. On analyzing the strength of the association between the observed disease burden and obesity-related coverage, we found that cardiovascular diseases were the most frequent disease type. In comparison, metabolic dysfunction and inflammation caused by obesity are ignored in the news, despite scientific evidence indicating that metabolic dysfunction caused by obesity contributes to a wide variety of disorders and effects on the nervous system [33].

It is also clear that digital news frequently links the concept of obesity with NCDs such as “cardiovascular diseases,” “metabolic diseases,” or “cancer.” Current information circulates from the Chinese news serve as a wake-up call for the general public who have been slow to acknowledge that obesity is a disease and a dangerous disease paring with NCDs. Moreover, media content still emphasizes that obesity as merely a lifestyle issue or, worse, a moral failing due to lack of willpower. Thus, the communication or advice from the news reporting is usually just to eat less and exercise more which could be ineffective for most people with obesity, and those who do manage to lose weight tend to gain it back quickly. This is not a matter of will power but rather the body has evolved complex physiological processes to actively resist weight loss through metabolic, hormonal, and neurobehavioral changes [1-3].

Following a stronger focus on 2 shared causative attributes of obesity such as stress and tobacco use, another 3 causative inferences covering genetic disorders, overconsumption of alcohol, and decreased physical activity were also covered frequently. Four top causal inferences of obesity were about an individual’s responsibility in handling stress, tobacco, alcohol, and decreased physical activity in all newspapers. To be specific, the causative inferences of alcohol intake linked to obesity were prominent in Mainland China, while stress, tobacco use, and decreased physical activity received more media attention in Hong Kong and Taiwan. The pattern in showing various causal inferences can be understood as an indication of a feature distinctive of obesity communication in the area.

Consistent with the existing literature [1,2,6], the obesity epidemic—according to news reports—is increasing the burden of several NCDs such as heart disease, diabetes, and cancer. News reports advise that obesity is associated with poor health outcomes, increasing the likelihood of developing NCDs and conditions including obstructive sleep apnea syndrome. At first glance, one might think that chronic illnesses are sufficient to identify interventions for analyzing causal language and the strength of the effect of NCDs on obesity. More combined and rigid scientific interventions should be investigated for reporting in the news because both individual and social transmission of obesity is apparent; populations achieve obesity through some combinations of mechanisms such as lack of exercise, genetic disorders, unhealthy lifestyle, and a poor food environment. Thus, a classification of obesity to being socially communicable should be acknowledged in the news.

The state-owned media may have certain advantages relative to commercial-driven news that the health educator may want to consider. For instance, Mainland China’s state-owned media may have far greater reach of readership than business-oriented and private news organizations in Hong Kong and Taiwan. A market-driven newspaper such as Apple’s Daily News from Hong Kong linked casual reasoning most frequently between decreased physical activity and obesity, followed by genetic characteristics and obesity concerns. In contrast, United Daily News from Taiwan focused more commonly on fat mass and obesity-associated genes, followed by lack of physical activity, as causal attributes for obesity. Similarly, Guangzhou Daily from Mainland China linked obesity with genetic disorders, followed by the consequence of lack of physical activity.

This study aims to understand how themes and scenarios about the outcomes and implications of news coverage on obesity are reconstructed and acted upon. The substance of investigative news reports should demonstrate the claim that the contemporary digital news media is best characterized as a narrative short communication prepared on the topic of obesity and its potential communicability. We nowadays face new concentrations of power, leading to new inequalities and insecurities with respect to data ownership, data geographies, and data interpretation. Learning the coverage of the obesity epidemic will likely change practice for health researchers, journalists, and medical professionals, as shifts will be seen in the incidence of obesity-related disease severity.

### Comparison With Prior Work

The causal links in media texts can be made explicitly or implicitly. Over the past decade, numerous causal inferences and effects for interventions have been evaluated [3,8,10,21], but very little evidence exists about their long-term effectiveness. An algorithmic strategy can extract and classify complex semantic contents in lingual media discourse. Thus, millions of news texts at minimal added human effort afford researchers control over the process of theory-guided classification and with scientific findings.

Other viewpoints are consistent with previous reports. First, health news can play a positive role in promoting public health or a negative role in causing unnecessary fear [16,29]. The episodic coverage emphasized attributions of individualistic causal responsibility and the punitive treatment outnumbered the thematic coverage for increased societal attributions. The most severe issue with causal inferences of news reported was to blame individuals for their own health afflictions, but little consideration is given to larger social and environmental issues [5,6,8,16]. Thus, attributing obesity to a failure of personal responsibility may impair and limit social, economic, environmental, and governmental approaches for addressing it.

Second, Chinese news offered gene-based explanation of obesity disorder at the highest frequency (61.6% to 80.7%). It has been reported that news stories often misrepresent genetics by exaggerating the causal link between genes and obesity [34]. The single biomedical frame related to obesity issues in the news was highlighted, despite various causal factors that should be taken into consideration [8,34]. The news reports that offer gene-based explanations regarding obesity may make audiences believe that genetic influence is the primary cause of the obesity disorder, compared to news coverage that suggests that obesity is a consequence of one’s unhealthy lifestyle [34].

### Limitations and Challenges

Several limitations are noteworthy. The purpose of this study is to identify causal inference patterns in news coverage on obesity communication. There are challenges and limitations for categorizing a vast amount of news data and considering the news featured frame as mixed. In the changing scope of news reporting, significant issues within journalism as a professional field have emerged. Additionally, the readership complexity can be quite challenging in terms of suggesting interventions and fostering media education. The rapid diffusion of internet-based news on mobile devices has also created limitations for linking communication effects.

There are challenges with computational methods, including the lack of standards for automated content measures and their interpretation of information. Although the implicit causal inferences in this study may suggest or infer causation, the number of articles on obesity-related diseases may not be completely representative. These are all attributed to language complexity in similar research [9,32,34,37]. A variety of diseases can develop over the course of time; as a result, our findings limit the use of data for prediction and actions to improve the effectiveness of news education.

The experience gained with previous research and epidemiological problems such as obesity interventions should help expand the required health initiatives and increase the likelihood of preventing future generations from facing the consequences of obesity. Interventions including obesity communication is not straightforward but rather conditional on the confounders. As new features are being introduced and the programming languages continue to evolve, mixed methods such as advanced quantifying studies in data journalism, agent-based modeling, and experimental research of news study are suggested.

### Conclusions

This study examined journalistic work on the impacts and effects of excess weight and its potential links of etiology and diseases from 2010 to 2019. Our results show the coverage of obesity evolving over time, the path it takes, and the words and phrases that surround the topic. A total of 21 cancers and 11 behavioral factors are identified to be associated directly with overweight- and obesity-related communication. Overall, the increasing trend of coverage illustrates the interaction of journalistic and public health concerns in supporting obesity communication.

News content associated with a given unhealthy behavior is presented in more than half of the articles. Concomitantly, the NCDs associated with the obesity epidemic are also found frequent in over 60% of the articles. Two frames were considered for a useful discussion concluding that the framework of obesity involves individual-level rather than societal etiologies. A more prevalent frame in future studies is proposed because our findings suggest far more complexity than is apparent for discussion.

With the increasing amount of web-based news and the incorporation of computational methods, it is possible to extract meaning from a large volume of data and to apply computational algorithms to unstructured data. In particular, few studies have focused on the Chinese data, which suffer from a heavy underrepresentation from empirical and theoretical research. Nevertheless, this study moves media analysis beyond the realm of the traditional content analysis by gathering metadata to support and contradict our suspicions.

## Appendix

Multimedia Appendix 1 Obesity-related keywords in Chinese and translation in English.

Multimedia Appendix 2 Examples of conceptual terms in news media with English translation.

## Abbreviations

EC: edge centrality
NCD: noncommunicable disease
RCC: renal cell cancer
