# Supplementary material for: Obesity-Related Communication in Digital Chinese News From Mainland China, Hong Kong, and Taiwan: Automated Content Analysis
Source: JMIR Public Health Surveill. 2021 Nov 23;7(11):e26660. doi: 10.2196/26660 (PMC8663590; doi:10.2196/26660)

**Multimedia Appendix 1**

Appendix I. Obesity-related keywords in Chinese and translation in English.


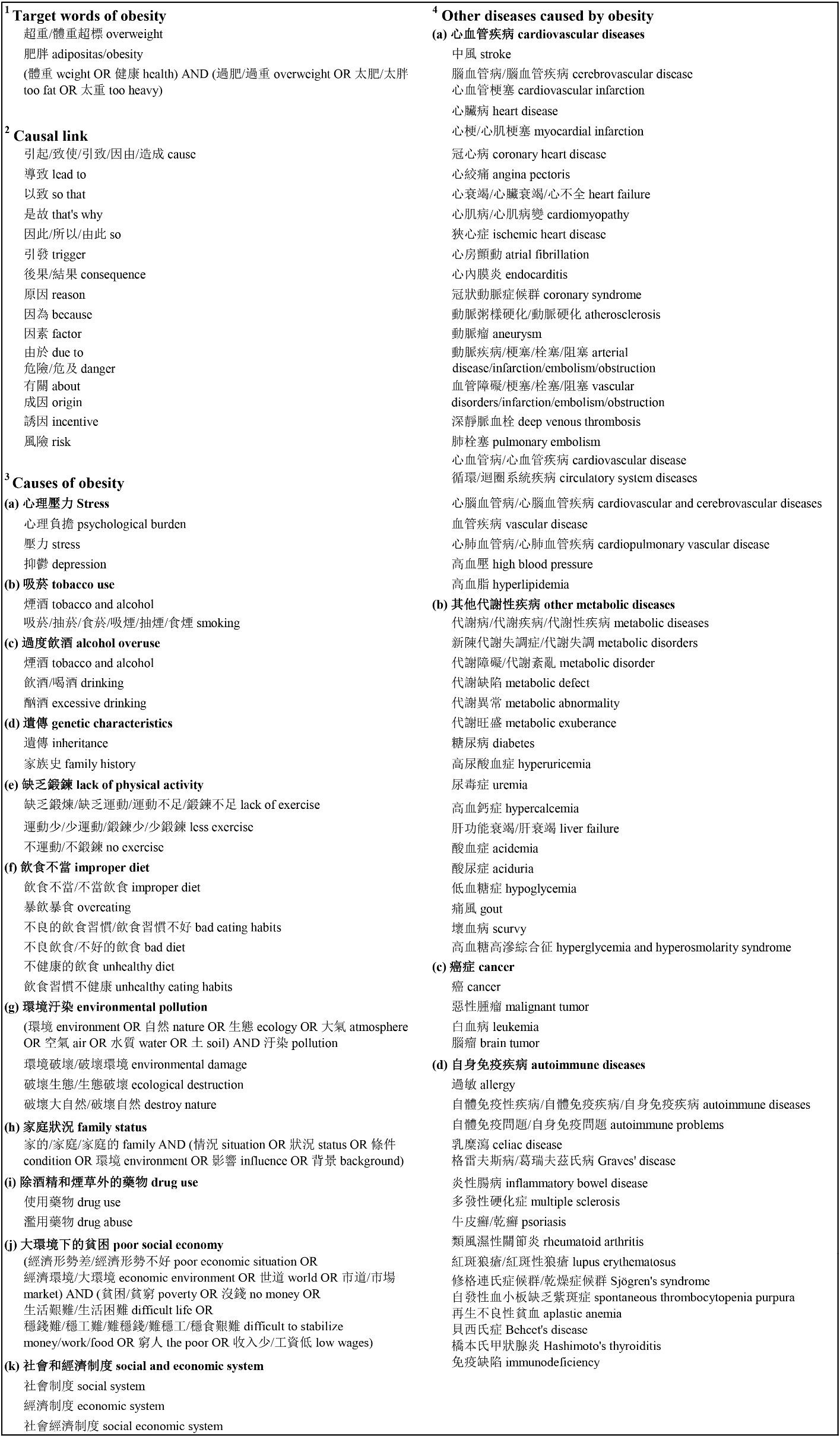

Supplement: Multimedia Appendix 1 [file publichealth_v7i11e26660_app1.docx]
