# Supplementary material for: Obesity-Related Communication in Digital Chinese News From Mainland China, Hong Kong, and Taiwan: Automated Content Analysis
Source: JMIR Public Health Surveill. 2021 Nov 23;7(11):e26660. doi: 10.2196/26660 (PMC8663590; doi:10.2196/26660)
Supplement: Multimedia Appendix 2 [file publichealth_v7i11e26660_app2.docx]

**Multimedia Appendix 2**

**Appendix II.** Examples of conceptual terms in news media with English translation.


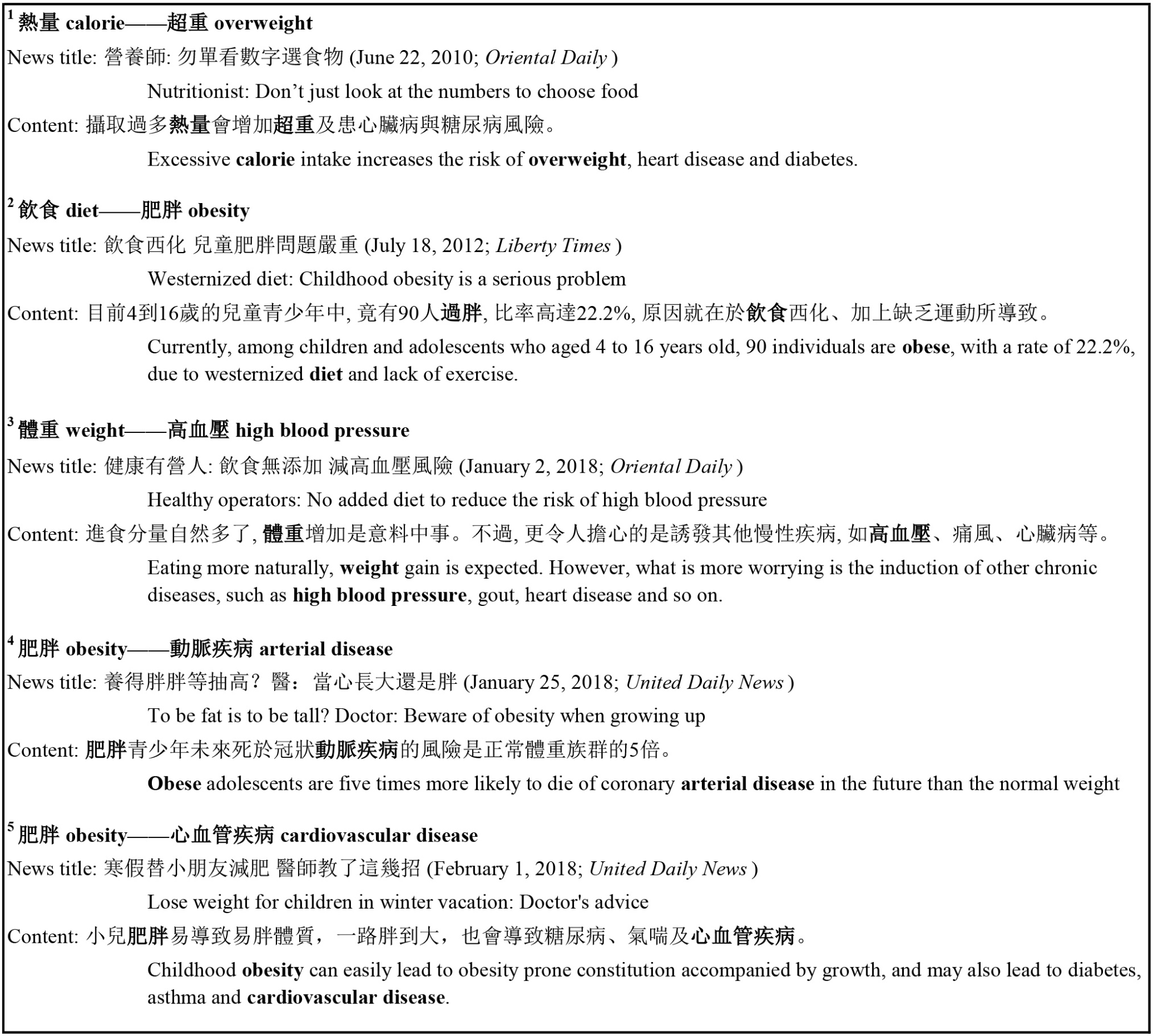


**Source Links of Five Examples**

1. 熱量 calorie——超重 overweight

<http://orientaldaily.on.cc/cnt/news/20100622/00176_098.html>

2. 飲食 diet——肥胖 obesity

<https://news.ltn.com.tw/news/life/breakingnews/668356>

3. 體重 weight——高血壓 high blood pressure

<https://orientaldaily.on.cc/cnt/news/20180102/00176_102.html>

4. 肥胖 obesity——動脈疾病 arterial disease

<https://health.udn.com/health/story/6057/2948953>

5. 肥胖 obesity——心血管疾病 cardiovascular disease

<https://health.udn.com/health/story/6057/2961973>
